# Evaluating the Use of Environmental DNA as a Method to Determine Occupancy and Distribution of Coeur d'Alene Salamanders in Montana

**DOI:** 10.1002/ece3.72888

**Published:** 2026-01-12

**Authors:** Jessica A. Coltrane, Torrey Ritter, Hannah Specht, Daniel H. Mason, Alissa Anderson, Thomas W. Franklin

**Affiliations:** ^1^ Montana Fish, Wildlife and Parks Kalispell Montana USA; ^2^ National Genomics Center for Wildlife and Fish Conservation USFS Rocky Mountain Research Station Missoula Montana USA

**Keywords:** Coeur D'alene salamander, detection probability, distribution, eDNA, occupancy, *Plethodon idahoensis*

## Abstract

Coeur d'Alene salamanders ( *Plethodon idahoensis* ) are found in small, isolated populations in the mountains of Montana, Idaho, and British Columbia. Effective conservation of this species hinges upon understanding population distribution and connectivity; however, identifying occupied Coeur d'Alene salamander (CDL) sites is challenging. Surveys in Montana have been haphazard and have involved biologists searching potential sites during rainy nights in the early summer. To evaluate alternative survey methods, we sampled water at known‐occupied CDL sites across western Montana to evaluate the efficacy and applicability of using environmental DNA (eDNA) to detect CDLs, as well as covariates that impacted CDL detection. We first developed a species‐specific eDNA Taqman quantitative PCR assay. We then used repeated eDNA sampling to evaluate how different environmental covariates would impact detection probability of CDLs and used these results to recommend a survey protocol. We found that optimal eDNA sampling conditions for CDLs occurred at night, within 50 m of the downstream extent of preferred salamander habitat, and at lower water flow rates. Under these conditions, five 5‐L water samples were required to achieve a detection rate above 79%. The results of this study revealed that eDNA analysis is a viable method to estimate CDL occupancy at potential sites across their range; however, eDNA analysis can be costly, so combining this method with visual searches is advised.

## Graphical abstract

The purpose of this project was to evaluate the efficacy of eDNA sampling to determine occupancy of Coeur d'Alene salamanders, an “imperiled” species in Montana. Previous methods used to identify occupied sites were haphazard with no measure of detection probability. Due to the unique habitat used by these salamanders (seeps and waterfall spray zones), it was unclear if eDNA sampling would be an effective tool. Our work suggests that indeed eDNA sampling is viable for determining occupancy, and we propose a protocol that may be employed to achieve a high rate of detection.

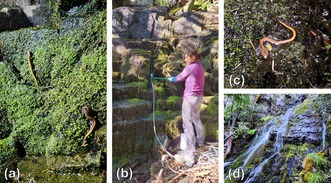

The Coeur d'Alene salamander ( *Plethodon idahoensis* ) is one of a few lungless salamander species found in the Northern Rocky Mountains of North America. They typically occupy temperate mesophytic forest types between 500 and 1500 m elevation in mountainous regions of northern Idaho (Slater and Slipp 1940), northwestern Montana, and southeastern British Columbia (Teberg 1964; Holmberg et al. 1984; Wilson et al. 1997). The southern extent of their range is delineated by the Selway River Drainage in Idaho and Sweathouse Creek in Montana (Wilson et al. 1997). In British Columbia, *P. idahoensis* is discontinuously found within the southern Columbia Mountains and within the Kootenai and Columbia River drainages. The northern limit is north of Revelstoke.

In Montana, Coeur d'Alene salamanders (CDLs) are listed as a Species of Concern (SOC) with a status rank of “Imperiled”, due to their limited range, small and potentially isolated populations, and high potential for statewide extirpation (MTFWP 2015). The Montana State Wildlife Action Plan (MTFWP 2015) further identifies these salamanders as a Species of Greatest Inventory Need (SGIN) and as a Species of Greatest Conservation Need (SGCN). Therefore, Montana Fish, Wildlife and Parks has prioritized monitoring and research efforts to better understand the distribution, status, and vulnerability of CDLs in Montana.

In Montana, the known distribution of CDLs is restricted to the far western part of the state (Figure 1) where CDLs are strongly associated with three primary habitats: springs and seeps, waterfall spray zones, and stream edges (Wilson and Larsen 1998; Werner and Reichel 1994; Wilson et al. 1997; Boundy 2001; Maxell 2002). As Montana's only lungless salamanders, CDLs have no aquatic larval stage. Adults and juveniles respire through their skin and are therefore restricted to damp, cool environments to avoid desiccation. During the day, individuals occupy rock crevices and other subterranean environments. On relatively warm, damp nights, they emerge to forage in wet leaf litter, moss, and rocks, usually within wet areas around their preferred habitat (Nussbaum et al. 1983; Wilson and Larsen 1998). These above‐ground forays are thought to be primarily restricted to spring and fall when environmental conditions reduce the risk of desiccation. During the heat of the summer and cold of the winter, CDLs primarily remain underground, and rare summer surface activities appear to be negatively correlated with high daytime temperatures and days since last rainfall (Wilson Jr. and Larsen Jr. 1988).

**FIGURE 1:**
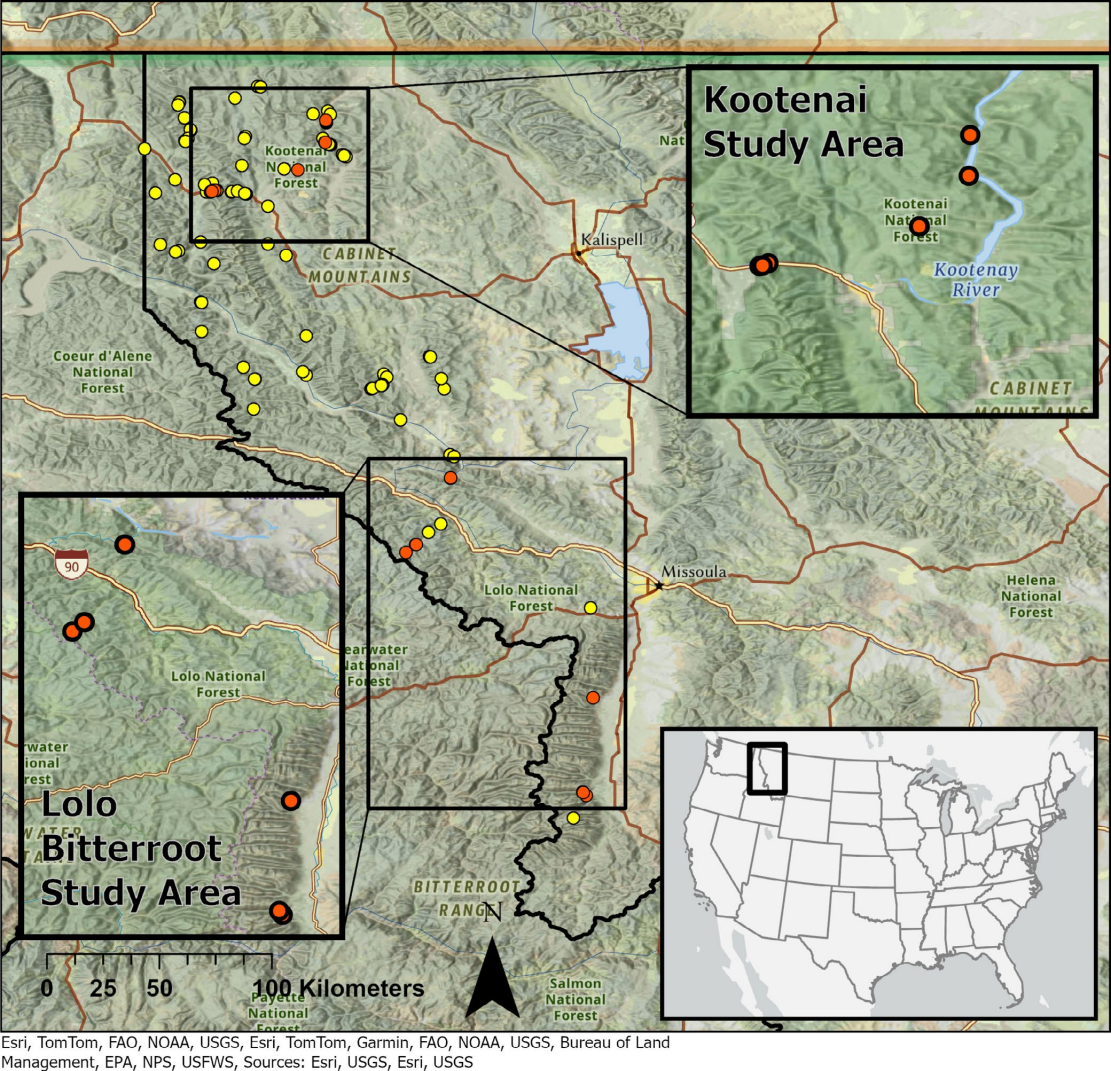
Observations of Coeur d'Alene salamanders in Montana, USA (1962–2020, Montana Natural Heritage Program; yellow dots) and Coeur d'Alene salamander eDNA study sites in the Kootenai, Bitterroot, and Lolo National Forests (2020–2022; orange dots).

CDL populations are thought to be small and isolated, but the extent of dispersal among adjacent sites is unknown. Movement overland likely requires heavy canopy cover and wet terrestrial conditions. These factors make CDLs vulnerable to local population extirpations from disturbances which may destroy or significantly alter habitat or directly kill individuals. Such threats include, but are not limited to, timber harvest, road and trail construction, water diversion, pollution, disease, predation, and wildfire (MTFWP 2015).

Effective conservation of CDLs hinges upon understanding the spatial distribution of populations and the amount of connectivity and gene flow among those populations. However, identifying occupied CDL sites and implementing long‐term monitoring of those sites has been challenging and haphazard. Survey efforts have historically been opportunistic and focused on rainy nights in early summer when surface air temperatures are above 10°C. Timing of surveys to coincide with warm but wet conditions is likely important to maximize detection but limits the number of surveys that can occur within a given year. Even under optimal conditions, it can take multiple observers several hours to locate even a single salamander. Furthermore, navigating to potential or known sites often requires long travel times through dangerous terrain in the dark. For these reasons, it can be difficult and inefficient to evaluate site occupancy with measurable confidence. To overcome these challenges and support conservation planning and monitoring, more efficient, sensitive survey methods are needed.

Environmental DNA (eDNA) has been used for over a decade in aquatic systems to detect and estimate relative abundance of target species and has proven especially useful for rare or threatened amphibian species that are difficult to observe (e.g., Olson et al. 2012, Thomsen et al. 2012, Pilliod et al. 2013, Rees et al. 2014, Walker et al. 2017; Preißler et al. 2019). By analyzing environmental samples for genetic material shed by organisms, survey methods using eDNA have successfully detected species in circumstances when other methods have not (Fediajevaite et al. 2021). However, the efficacy of using eDNA analysis to detect individual species varies due to differences in how species‐specific DNA is deposited and degrades in the environment (Lamb et al. 2022)

We collected water samples at known‐occupied CDL sites across western Montana to evaluate the efficacy and applicability of using eDNA analysis to detect CDLs. Our overall goal was to design a sampling protocol to assess CDL distribution that could be incorporated into long‐term monitoring for site occupancy. Specifically, our first objective was to develop a sensitive and specific assay targeting a region of the mitochondrial *cytochrome b* (cytb) gene in CDLs and then use this assay to determine whether CDLs could be detected in the field using eDNA sampling. Our second objective was to evaluate how survey timing (time of year, time of day, time after rain events) and site conditions, such as water flow rate and distance from CDL habitat, impacted detection rate. Our third objective was to determine the number of water samples (volume of water) required to detect CDLs with a high degree of confidence given site conditions. We hypothesized that the highest detection rate would occur closest to the area of CDL activity and at night when CDLs were active above ground. We assumed detections would be highest within 48 h of rain events, when sites would be wet enough to facilitate salamander movement. We also hypothesized that detection rates would be higher during periods with lower flow rates due to dilution effects of high flow on eDNA (Curtis et al. 2021). We assumed low availability of eDNA at each site, and therefore we suspected that multiple water samples would be needed to determine whether a site was occupied with a high degree of certainty.

## 1 Study Area

This study was conducted at known‐occupied CDL sites in western Montana in the Kootenai, Bitterroot, and Lolo National Forests (Figure 1). The Kootenai National Forest falls within the North Central Rockies Forest ecoregion (Olson et al. 2001) and is characterized by warm, moist summers and wet, snowy winters. Precipitation in the Kootenai National Forest averaged 25.4–101.6 cm annually, depending on location, with the highest amounts of precipitation falling in November and December. Mesic plant species more typical of the Pacific Northwest resided alongside those of drier inland habitats (Cooper et al. 1991), producing one of the most biologically diverse regions in Montana (Pfister et al. 1977; Leavell 2000). Cottonwood ( *Populus trichocarpa* ), aspen ( *Populus tremuloides* ), and willow (*Salix* spp.) dominated the river bottoms, while eastern red cedar ( *Thuja plicata* ), grand fir ( *Abies grandis* ), and western hemlock (*Tsuga hetrophylla*) occupied moist, low‐elevation drainages. Mixed upland forests composed of Douglas‐fir ( *Pseudotsuga menziesii* ), lodgepole pine ( *Pinus contorta* ), and western larch ( *Larix occidentalis* ) were found in mid‐elevation areas. Wet, high‐elevation sites were dominated by subalpine fir ( *Abies lasiocarpa* ), Engelmann spruce (*Picea engelmanni*), and mountain hemlock ( *Tsuga mertensiana* ). Ponderosa pines ( *Pinus ponderosa* ) were restricted to xeric sites often on southern or eastern aspects (Vinkey 2003).

The Lolo National Forest and the Bitterroot National Forest are oriented generally north–south along the Montana‐Idaho border and represent a transition zone from the more mesic forests of northwest Montana and the drier forests of southwest Montana. Both National Forests are within the Northern Rockies ecoregion. The climate was predominantly continental, experiencing significant temperature variations throughout the year, with average winter temperatures ranging from approximately −9°C to 4°C, and summer averages from 11°C to 28°C (Belote and Aplet 2014). Annual precipitation averaged 75–125 cm, depending on specific microclimates influenced by elevation and vegetation. Dominant streamside vegetation included cottonwood ( *Populus trichocarpa* ), aspen ( *Populus tremuloides* ), willow (*Salix* spp.), alder (*Alnus* spp.), Rocky Mountain maple ( *Acer glabrum* ), and red‐osier dogwood ( *Cornus sericea* ). Dominant upland vegetation generally reflected that of the Kootenai National Forest, but moist forest types were more restricted to valley bottoms and wetter microsites than in the Kootenai National Forest. The Bitterroot National Forest represents the presumed southern extent of CDL distribution in Montana (MTNHP and FWP 2025).

**FIGURE 2:**
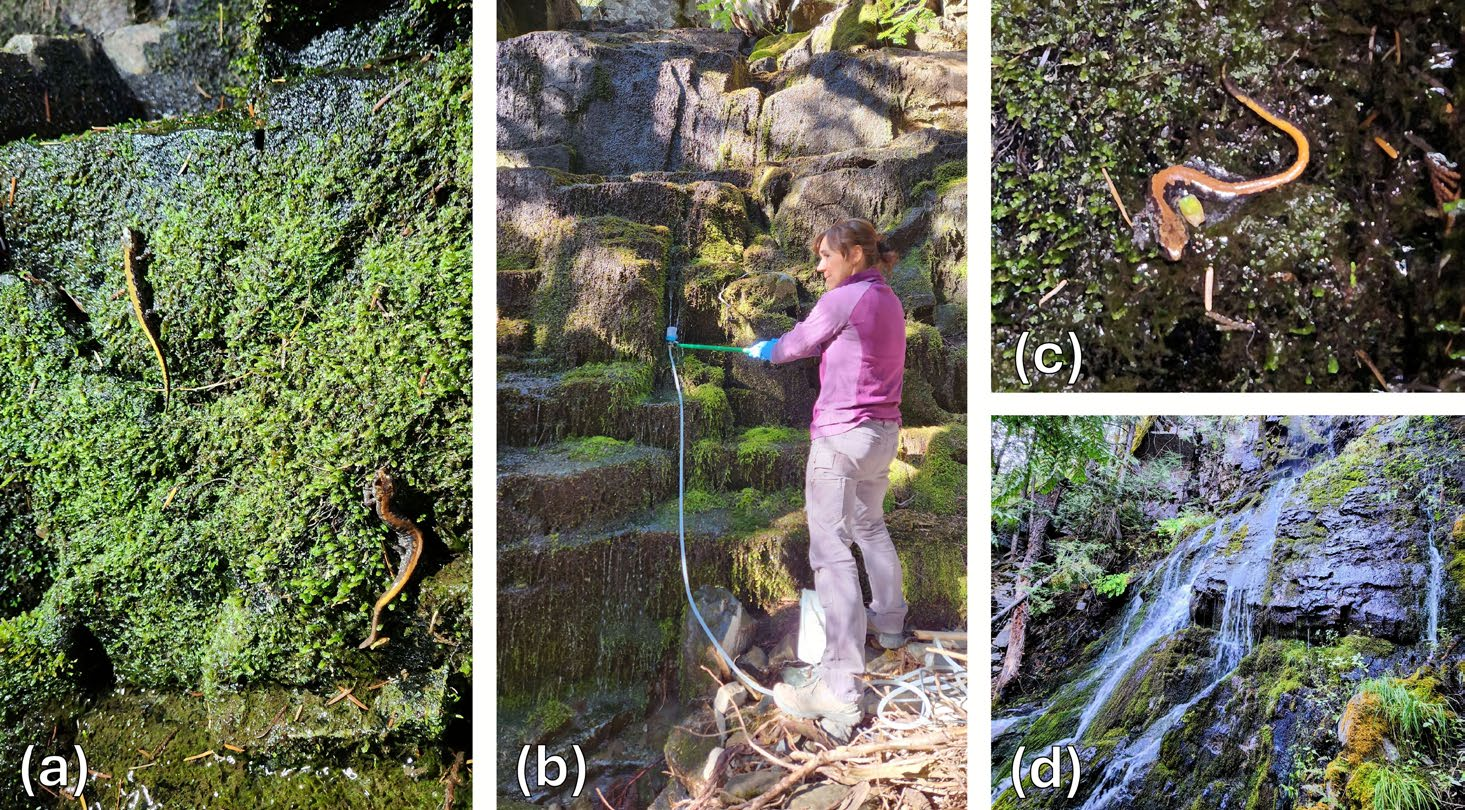
(a) Two Coeur d' Alene salamanders along a seep site after dark, (b) collection of eDNA from a seep site by J. Coltrane, (c) a Coeur d'Alene salamander, and (d) a waterfall site (bottom) in Montana, USA.

Typical occupied CDL sites across the study area were between 172 and 745 m in elevation and included moss‐covered seeps along fractured cliff faces and waterfall spray zones that supported persistent moss (Figure 2). Other amphibians found in the study area included long‐toed salamanders ( *Ambystoma macrodactylum* ), Columbia spotted frogs *(Rana luteiventris)*, western toads ( *Anaxyrus boreas* ), Pacific tree frogs ( *Pseudacris regilla* ), and Rocky Mountain tailed frogs ( *Ascaphus montanus* ).

## 2 Methods

This project was conducted in three phases: (1) We confirmed active CDL sites through visual encounter surveys; (2) We developed an assay and determined if eDNA analysis could be used to detect the presence of CDLs and which covariates may impact detection probability; and (3) We used the results from Phases 1 and 2 to develop a protocol to determine site occupancy with high confidence that could be used to assess the occurrence of and monitor CDL populations across their range. For our study, a “site” refers to a distinct portion of a stream drainage where habitat conditions are suitable for CDLs (e.g., a waterfall section along a stream or seep in a fractured rock face). Independent sites were defined as being greater than 200 m apart with no surface water connectivity among the sites.

### 2.1 Visual Encounter Surveys

We conducted night searches at 14 sites historically occupied by CDLs to determine occupancy. These sites were chosen as a representative sample of the variety of sites (both historic and potentially new) found in Montana. Sites varied in type and physical characteristics and number of salamanders found in previous searches (MTNHP unpublished data). Typically, only 1–3 individual salamanders are found at each site during a survey, which may suggest low abundances. We searched sites during spring and fall of 2022 and 2023 with the goal of identifying known‐occupied sites to assess the efficacy of eDNA sampling (Table 1). Once a site was determined to be occupied, no additional directed visual searches were conducted. We restricted searches to nights when ambient temperatures exceeded 7°C. Using headlamps, we searched areas surrounding seeps and springs and within waterfall spray zones, beginning 30 min after sunset and continuing for at least 2 h or until all suitable habitat at the site had been searched or salamanders were detected. We searched surface areas, as well as under rocks, moss, and logs. All features that were moved during search efforts were immediately replaced to avoid extensive site disturbance. A site was defined as occupied if at least one CDL was detected. When at least one CDL was located, we recorded a detailed description of the site including the characteristics of the rock, water flow rate, presence of moss, dominant vegetation, and environmental factors, such as recent fire and timber harvest (Table 1). We also noted the downstream extent of where CDLs were detected and available habitat at the site to determine distances from the occupied site for eDNA sampling.

**TABLE 1:** Description of study sites for Coeur d' Alene salamander eDNA sampling.

National forest	Site	Site type	Predominant rock type	Water source	Dominant vegetation	Moss coverage (%)	Evidence of fire	Timber harvest	Salamanders detected with eDNA
Kootenai	CD3	Waterfall	Fractured cliff/cobbles/boulders	Permanent	Mixed conifer forest	75–100	None	None	Yes
Kootenai	CD4	Seep	Fractured cliff	Permanent	Mixed conifer forest	75–100	None	None	Yes
Kootenai	CD5	Seep	Fractured cliff	Permanent	Mixed conifer forest	50–75	None	None	Yes
Kootenai	CD19	Seep	Fractured cliff	Seasonal	Mixed conifer forest	< 5	None	None	No
Kootenai	CD33	Seep	Fractured cliff	Seasonal	Mixed conifer forest	50–75	None	None	Yes
Kootenai	CD38	Waterfall	Fractured cliff/cobbles/boulders	Permanent	Mixed conifer forest	75–100	None	Heavy Harvest Adjacent	Yes
Kootenai	CD6	Seep	Fractured cliff	Permanent	Mixed conifer forest	75–100	None	None	No
Kootenai	CD8	Seep	Fractured cliff	Permanent	Mixed conifer forest	75–100	None	None	Yes
Bitterroot	LRC	Waterfall	Fractured cliff	Permanent	Mixed conifer forest	50–75	None	None	Yes
Lolo	TCS	Seep	Fractured cliff/scree	Permanent	Shrub	25–50	Within 50 years	None	Yes
Lolo	NFTCS	Seep	Fractured cliff	Permanent	Mixed conifer forest	25–50	None	None	Yes
Lolo	CCF	Waterfall	Fractured cliff	Permanent	Mixed conifer forest	75–100	None	None	Yes
Bitterroot	RC	Seep	Fractured cliff	Permanent	Mixed conifer forest	25–50	Within 50 years	None	No
Bitterroot	SF	Waterfall	Fractured cliff	Permanent	Mixed conifer forest	5–25	Within 50 years	None	No

### 2.2 Evaluating Efficacy of eDNA and Impact of Covariates on Detection Rate

#### 2.2.1 Assay Design and In Silico Assessment

To evaluate the potential of eDNA methods for CDL detection, we designed and validated a Taqman quantitative PCR (qPCR) assay targeting a region of the mitochondrial cytochrome b (cytb) gene. We downloaded cytb sequence data available from GenBank (*n* = 95) and aligned data in MEGA 7 (Kumar et al. 2016) to select a region of cytb which contained a sequence that was unique to CDL and therefore would create an assay with high fidelity to CDL eDNA and low probability of amplifying eDNA from nontarget species. The resulting CDL assay amplifies a 92‐base pair long region of the cytb gene using a probe with a FAM‐labeled and minor‐groove‐binding, nonfluorescent quencher (MGB‐NFQ) attached with two forward primers and two reverse primers (Table 2). Annually mixing the primers allowed us to accommodate single nucleotide polymorphisms (SNPs) within CDL without creating undesirable primer melting temperatures (*T*_m_) by adjusting primer length as well as primer sequence. We ensured that primer and probe *T*_m_'s, percent bases which were either guanine or cytosine, and lengths met recommended values using Primer Express 3.0.1 (Life Technologies) before assessing the characteristics of secondary oligonucleotide structures with the OligoAnalyzer web application (Integrated DNA Technologies).

**TABLE 2:** Primer and probe sequences, amplicon length, and optimal concentrations for the Coeur d'Alene salamander assay.

Assay component	Sequence (5′‐3′)	*T*_m_ (°C)	Amplicon length (nt)	Optimal concentration (nM)
Forward A	TGTTCTCCCATGAGGACAAATGT	59.3	92	450
Forward B	TGTTCTCCCATGACGACAAATG	59.4		450
Reverse A	TGTACAATGGTGTCTCCTATATATGGAATT	59.3		450
Reverse B	TGTACAAGGGTTTCTCCTATATATGGAATT	59.7		450
Probe	FAM‐TAATCACTAACCTACTCTCCG‐MGBNFQ	70		250

We examined the potential for the CDL assay to amplify eDNA from nontarget species (Table 3) in silico with specificity predictions produced using eDNAssay, a machine learning tool designed for evaluating eDNA assays (Kronenberger et al. 2022). The input alignment included the CDL assay and cytb data representing 329 of the currently estimated 476 plethodontids known from North America (Appendix A). We applied a conservative assignment probability threshold of 0.3 to identify nontarget species which possibly could produce qPCR amplifications, and a threshold of 0.5 to identify species which probably would produce amplifications (Kronenberger et al. 2022).

**TABLE 3:** Species tested and results for tissue‐derived DNA samples used during in vitro testing for taxonomic specificity of the Coeur d'Alene salamander assay. Origin refers to the US state or Canadian province of collection for tissue DNA. *N* refers to sample size. “+” indicates that we visualized amplification with the assay; “−” indicates we did not visualize amplification.

Family	Species	Common name	Origin	*n*; amp result
Plethodontidae	*Plethodon idahoensis*	Coeur d'Alene salamander	ID	10; +
Ambystomatidae	*Ambystoma macrodactylum*	Long‐toed salamander	ID	4; −
	*Ambystoma gracile*	Northwestern salamander	AK	4; −
Salamandridae	*Taricha granulosa*	Rough‐skinned newt	AK	8; −
Ascaphidae	*Ascaphus montanus*	Rocky Mountain tailed frog	MT	1; −
	*Ascaphus truei*	Coastal tailed frog	OR	2; −
Bufonidae	*Anaxyrus boreas*	Western toad	MT, WY, ID	1, 2, 2; −
Hylidae	*Pseudacris maculata*	Boreal chorus frog	WY	2; −
	*Pseudacris regilla*	Pacific tree frog	CA	3; −
Ranidae	*Lithobates catesbeianus*	American bullfrog	CA	2; −
	*Rana luteiventris*	Columbia spotted frog	MT, WY	1, 1; −
	*Rana aurora*	Northern red‐legged frog	CA, OR	1, 2; −
	*Lithobates sylvaticus*	Wood frog	AK, Saskatchewan	1, 1; −

#### 2.2.2 Experimental Assay Validation

Experimental assay validation experiments were performed in lab space dedicated for the purpose, separate from areas of the NGC committed to eDNA sample analysis, end‐point PCR, or genomic sequencing experiments. To identify the optimal concentration of the assay primers, we tested 16 unique combinations (each at 100, 300, 600, and 900 nM) in triplicate reactions in a single qPCR experiment on a QuantStudio 3 qPCR System (Life Technologies). The probe concentration was held constant at 250 nM. qPCR occurred in 15 μL reactions with 7.5 μL of Environmental Master Mix 2.0 (Life Technologies), 3.5 μL of 20X primer and probe mixture at the primer concentrations which were being tested, and 4 μL of CDL genomic DNA, diluted to 0.1 ng μL^−1^ (Dysthe et al. 2018). A triplicate control was included to test for cross‐contamination of the qPCR reagents and allow for the effect of assay concentration to be isolated in our experiment; the control replaced the 3.5 μL of premixed assay with an equivalent volume of deionized water. CDL genomic DNA, as well as DNA used for in vitro testing against nontarget species (Table 3), was extracted from tissue samples in accordance with MFWP regulations and in a manner consistent with ethical and permit guidelines. Tissue samples were stored either in 95% ethanol or dried onto chromatography paper for transport to the laboratory. To reduce the risk of any cross‐contaminating DNA being introduced into the extraction, we rinsed each tissue with a 10% sodium hypochlorite solution, then rinsed it twice with deionized water before we extracted genomic DNA using a DNeasy Tissue and Blood Kit (Qiagen Inc.) following the manufacturer's protocol. We quantified DNA from the samples with a Qubit 2.0 fluorometer (Life Technologies) prior to dilution in TE buffer (Integrated DNA Technologies). Extracts were stored frozen at −30°C until experiments could be completed. All qPCR reactions used a simple profile of 95°C for 10 min, followed by 45 cycles of denaturation at 95°C for 15 s and annealing and extension at 60°C for 1 min. We used single‐replicate qPCR reactions to confirm the assay's performance in vitro with these CDL tissue DNA samples and likewise evaluated its specificity in vitro against tissue DNA from a set of 12 nontarget amphibian species which could co‐occur with CDL across their range (Table 3).

Once we determined the optimal primer concentrations for the CDL assay, we cleaned qPCR product from the optimization experiment with a GeneJET PCR Purification Kit (ThermoFisher), quantified the products with a Qubit, and prepared a standard curve with a five‐fold serial dilution. This resulted in a curve with concentrations of 31,250, 6250, 1250, 250, 50, 10, and 2 copies reaction^−1^, which we tested using the optimized assay in six replicates each, alongside a six‐replicate, no‐template control (NTC), on a single qPCR plate. We used the results of this experiment to estimate the CDL assay's limit of detection (LOD) using the method of Bustin et al. (2009), which simply defines LOD as the lowest concentration for which at least 95% of the replicates amplify.

As a comprehensive test of the filtering, eDNA extraction, and qPCR analysis with the CDL assay, we tested six eDNA samples collected using the procedure of Carim et al. (2016) at five locations which were known to support CDLs. These sites were additional to the field sites described in the remainder of the study.

### 2.3 Field Collection of eDNA Samples

During September–October 2022, June–July and October 2023, and June and October 2024, we collected eDNA samples at 14 sites where CDL presence had been confirmed with visual surveys; 2 additional sites where CDLs had been observed in visual surveys were excluded due to lack of surface water available for eDNA sampling. We used methods outlined in Carim et al. (2016) to collect water samples to be analyzed for the presence of CDL eDNA. Per this protocol, we did not collect distilled water controls at each site; however, positive and negative control samples were included in each laboratory analysis to ensure that contamination did not influence the sample results. For each sample, we filtered 5 L of water using a portable electric peristaltic pump (series II Geotech Geopump). We used a 3.7 s/100 mL flow rate, Grade 934‐AH, 47 mm diameter Whatman 1827‐047 Glass Microfiber Binder‐Free Filters with a particle retention capacity of 1.5 μm (Thermo Fisher Scientific Inc.).

To evaluate the best practices for timing sampling and for collecting samples relative to site characteristics, we explored the effects of time of day (day vs. night and time after sunset), season (spring vs. fall), water flow rate, time after rain event, and distance from site on eDNA detection probability. Flow rate was categorized using the following descriptors based on visual estimation: garden hose (0–850 L/min), firehose (850–2548 L/min), 10‐in. culvert (2548–11,893 L/min), 30‐in. culvert (11,893–110,436 L/min), and large stream (≥ 110,436 L/min). To determine the optimal sampling time frame, we sampled each site during the spring/early summer and in the fall, since CDL surface activity may vary by season; however, some sites did not have surface water in the fall and were therefore unavailable for sampling during that time. During each site visit, we collected 1–5 water samples at the “origin point” of each site. Origin points were 0–1 m from the downstream extent of habitat assumed to support CDLs (i.e., stream or waterfall spray zones, and moss‐covered areas surrounding seeps). We reasoned that locations closest to the origin point would have the greatest concentrations of eDNA, and additionally that collecting many samples close to the origin point would provide power to assess seasonal and diurnal timing of sampling. To evaluate if salamanders' nighttime surface activity impacted detection rate, we collected paired samples during daylight hours and at least 30 min after sunset (onset of darkness) within a 24‐h period at each site.

To evaluate how the proximity of an eDNA sample to salamander habitat affected detection rate, we collected samples at predefined increments downstream of the origin point. When possible, we sampled at the origin point (0–1 m), as well as at 5, 15, 30, and 50 m from the origin point. We initiated sampling at the furthest downstream distance, working our way to the origin point to avoid sample contamination. We collected additional samples at further distances from the origin point when possible. Due to the variability in water flowing downstream from the origin point among sites, the total number of samples collected at each site varied among sites and sometimes across seasons. To estimate detection probability, all sites were visited for sampling at least three times with a minimum of 24 h separating replicate eDNA sampling events. Finally, we collected samples across a range of streamflow levels, which varied both among sites and within sites at different sampling periods.

### 2.4 eDNA Analysis

All samples were sent to the NGC within a week of collection (National Genomics Center, United States Forest Service Rocky Mountain Research Station, Missoula, Montana, USA) for eDNA analysis. Upon receipt of samples at the NGC, sampling data were cataloged, and samples were stored at −20°C until analysis was conducted. All experimental work took place in a laboratory space dedicated for low‐concentration DNA handling and preparation, and no materials or reagents from high‐concentration DNA lab spaces were used or stored in proximity to samples or eDNA equipment. For each sample, eDNA was extracted from half of the sample filter using the Qiagen DNEasy Blood and Tissue Kit following a modified protocol described in Franklin et al. (2019). The other half of the filter was retained and stored at −20°C. If a sample required multiple filters, DNA from all extracted filter halves for a given sample was combined during DNA extraction.

All samples were analyzed using a species‐specific and sensitive quantitative PCR (qPCR) assay developed by the NGC to detect a region of the CDL mitogenome as described previously. Each sample was analyzed in triplicate (Franklin et al. 2019) on a QuantStudio 3 qPCR System (Life Technologies) alongside a triplicate positive and negative control. Since it is not necessary for the amount of eDNA in a sample to be greater than the LOD in order for it to be detected, a sample was considered positive for the presence of the target species if one or more of the three qPCR reactions amplified DNA of that species.

All reactions included an internal positive control to ensure that the reaction was effective and sensitive to the presence of CDL DNA. If the internal positive control appeared inhibited (i.e., reduced amplification of the control DNA due to chemical compounds in the sample), the sample was treated with a PCR inhibitor removal kit (Zymo Research) and re‐analyzed in triplicate. Removal of inhibitors may result in loss of DNA in a sample; however, with elution volumes of 100–200 μL, loss of DNA during inhibitor removal is on average less than 10% (see http://www.zymoresearch.com for more details). Thus, to minimize potential DNA loss in samples treated for inhibition, the second half of the sample filter was extracted and all extracted DNA from a given sample was combined to obtain ~200 μL of extracted DNA. If inhibition persists after treatment with the inhibitor removal kit, the sample was further treated through dilution and re‐analyzed. Diluting the amount of DNA in a PCR by increasing the ratio of water to DNA may reduce the effects of inhibitors in PCR analysis (McKee et al. 2015). If a sample was diluted, the number of replicates was also increased to 12 to keep the total amount of DNA analyzed consistent. All laboratory steps were conducted with negative controls to insure there was no contamination during kit assembly, DNA extraction, or qPCR setup.

### 2.5 Statistical Analysis

We used generalized linear mixed effects models with a logit link for analyses assessing variation in detection of CDL DNA relative to sampling conditions, with models implemented in R Studio (RStudio 2020. Version 2023.12.1) using package glmmTMB (Brooks et al. 2017), with effects plotted using ggplot and ggeffects (Wickham 2016; Lüdecke 2018). Wald tests were used to interpret coefficient significance (per use of glmmTMB package; Brooks et al. 2017). We ran separate models to address individual questions (Table 4) related to conditions and timing of sampling (Table 5) using subsets of samples collected to address each specific question (subset descriptions included in Table 4). An occupancy model was not required to examine detection variability because all sites were known to be occupied. We collected samples to provide adequate power to address each question. Since all variables explored were categorical, including all samples and all variables in a single model would have reduced power to address individual hypotheses. For example, when assessing the effect of sample distance from the origin point on detection, we only included samples from the origin point from the same day that distance samples were taken to eliminate unmeasured variation from samples taken on days when distance samples were not taken. Running different models instead of one global model to address these hypotheses carries a higher risk of a false positive result; we accepted this potential consequence which would translate to a more targeted temporal application of sampling where it might not be warranted, something which would be unlikely to impact the status of the species. Based on the model findings, we estimated detection probability from the subset of samples collected under the best conditions and two different flow rates and, from this, calculated the number of 5‐L water samples taken from a single location needed to assess CDL occurrence with different levels of confidence under a variety of conditions, based on calculated cumulative detection probability.

**TABLE 4:** Descriptions of the generalized linear mixed models used to evaluate the effects of different sampling conditions and strategies on the probability of detecting Coeur d'Alene salamander using eDNA in Montana, USA.

Question	Model structure [*data subset*]	Sites (*n*)	Samples (*n*)
Does daytime (day vs. night) impact detection rate?^a^	Night + (1|Site‐Date) [*sites with paired day and night samples*]	13	387
Does the amount of time after sunset impact detection rate?	Hour after Sunset + (1|Site) [*samples collected at or after sunset*]	13	162
Does season impact detection rate?	Season + Night + Season * Night (1|Site) [*sites with both spring and fall samples*]	7	339
Does distance from origin point impact detection rate?	Distance from origin + Night +Distance * Night + (1| Site‐Date) [*sites where distance samples were taken*]	6	244
Does rainfall impact detection rate?	Time since rain + (1|Site) [a*ll data*]	14	420
Does water flow rate impact detection rate?^b^	Flow rate^a^ [*Excluded discharge level 3, with samples collected within 50 m*]	14	403
How many 5 L samples need to be taken to achieve a detection probability of > 0.75 under optimal sampling conditions and different flow rates?	~1 [*samples collected within 50 m, at night, at discharge*<*fire hose*]	10	123
	~1 [*samples collected within 50 m, at night, at discharge similar to a 10″ or 30″ culvert*]	5	60

^a^ We had a similar number of samples collected both day and night, so we did not include a categorical variable to account for potential differences between day and night.

^b^ Flow rate varied primarily across sites, so we did not include a site random effect in this model.

**TABLE 5:** Description of the factor variables used to evaluate the probability of detecting Coeur d'Alene salamander eDNA at known‐occupied sites across Montana, USA.

Variable	Definition
Night	Describes whether sample taken before or after sunset (day = 0, night = 1)
Site‐Date	Describes the unique group of data collected at the same site in the same 24 h period (which could include an afternoon and into the following early morning hours)
Hour after Sunset	Describes which hour after sunset sample was taken (0–60 min = 1st hour, 61–120 = 2nd hour…241–300 = 5th hour)
Site	Describes the unique group of data collected at the same site
Season	Describes spring or fall data collection
Distance from origin	Describes the distance at which a sample was collected from the nearest known CDL location based on visual surveys. (0‐1 m, 5 m, 15 m, 30 m, 50 m)
Night	Describes whether sample taken before or after sunset (day = 0, night = 1)
Time since rain	Describes timing of last rain event relative to sampling (< 24 h, 24–48 h, > 48 h)
Flow rate	Describes discharge volume across 4 levels (Garden hose, fire hose, 30″ culvert, Large Stream); an additional level (10″ culvert) was excluded due to paucity of data

## 3 Results

### 3.1 Experimental Assay Validation

**FIGURE 3:**
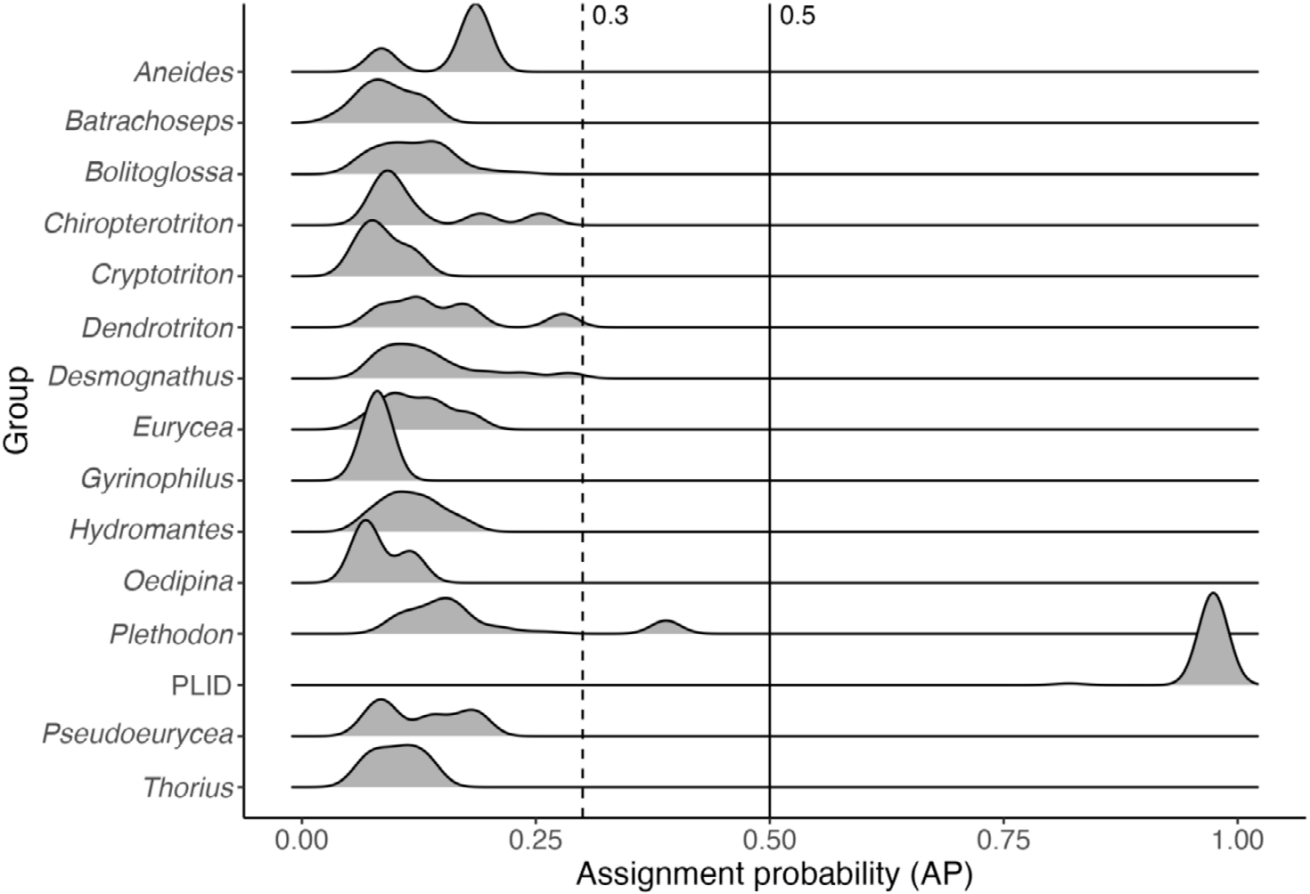
Density plot of the modeled probability of nontarget plethodontids' cytb sequences being assigned to the amplify class (AP), produced with eDNAssay (Kronenberger et al. 2022). Results for species were grouped into genera except for the target taxon ( *Plethodon idahoensis* ), which is grouped as “PLID.” Nine genera which were represented by only one species in the cytb data were omitted from the plot because their density amplitudes were too low to visualize. An assignment probability threshold of 0.3 is marked by a dashed vertical line; one nontarget species of Plethodon produced APs > 0.3 ( *P. vandykei* ). An assignment probability threshold of 0.5 is marked by a solid vertical line. Only the target species' APs exceeded this threshold. The complete eDNAssay output is available as a Supporting Information.

The assay amplified DNA from individual CDLs tested, and we failed to observe any amplification of DNA from the selection of nontarget amphibian species we tested in vitro (Table 3). qPCR with optimal primer concentrations (450:450:450:450 nM, Table 2, Figure 3) amplified a standard curve with good efficiency (Slope = −3.576, y‐int = 39.682, *R* ^2^ = 0.992, Efficiency % = 90.396), and produced an estimated LOD of 10 copies reaction^−1^ (6/6 replicates amplified). Four of 6 replicates amplified at 2 copies reaction^−1^, suggesting that the CDL assay had the sensitivity required to produce accurate eDNA detections. The results from in situ testing of the 6 eDNA samples from the 5 sites known to support CDLs suggested that the overall sensitivity of eDNA sampling depended on more than merely assay sensitivity; we detected CDL eDNA in only 3 of the 6 known positive water samples.

### 3.2 Assessment of Factors Affecting Detection Probability

We collected 430 water samples at 14 sites that were analyzed for CDL DNA. Seventy‐five of those samples yielded positive DNA results from 10 sites, and 6 samples were not viable. Samples from 4 sites visually verified as having CDLs did not test positive for CDL DNA.

**FIGURE 4:**
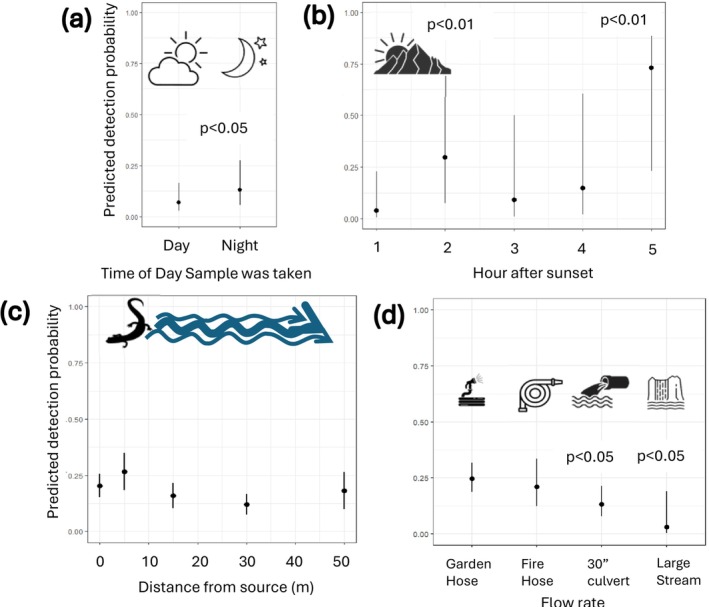
Estimates of predicted detection probability with 95% confidence intervals for occurrence of Coeur d' Alene salamanders relative to sample collection conditions: (a) day or night sampling, (b) time since sunset, (c) distance from origin point (area of greatest concentration of Coeur d'Alene salamander and/or optimal habitat conditions), and (d) estimated stream flow. *p* values represent values from Wald tests (per glmmTMB package; Brooks et al. 2017).

Our analyses indicate that samples collected at night have approximately twice the probability of detecting CDLs compared to samples collected during the day (Wald test, *p* = 0.045, day = 0.07 (95% CI 0.03–0.16), night = 0.13 (0.06–0.27); Figure 4). We conducted a post hoc analysis to determine whether there was an optimal time‐period after sunset during which to sample, since timing of night sampling varied among sites, spanning from 30 min to 5 h after sunset. A model using the subset of samples taken after sunset with a random effect of site (162 samples from 13 sites) showed that detection probability was worst in the first hour after sunset (0.04, 95% CI: 0.01–0.23), peaked in the second and fifth hours after sunset (2nd hour: 0.30, 95% CI: 0.08–0.69; 5th hour: 0.73, 95% CI: 0.23–0.96; *p* < 0.01) and was low and approximately equal in the third and fourth hours (3rd hour: 0.09 (0.01–0.50); 4th hour: 0.15 (0.02–0.60); Figure 4). We found no difference in detection probability based on the time since last rainfall (< 24 h: 0.13 (0.05–0.30); 24–28 h: 0.08 (0.02–0.25); > 48 h: 0.17 (0.08–0.33)). Season (spring vs. fall) did not uniformly impact detection probability across sites (Wald test, *p* = 0.35); spring sampling was better at 4 sites, while fall sampling was better at 3 sites. The optimal season for sampling at each site was most likely due to individual site conditions, primarily flow rate and salamander activity; however, we were unable to evaluate this with our dataset. We found that samples collected from 5 to 50 m from the origin point exhibited probabilities of detection that did not differ from that of the origin point (origin point detection probability = 0.13, 95% CI:0.04–0.34; Figure 4). We were not able to draw inference from the small number of samples collected beyond 50 m. Finally, we found a higher rate of detection at lower flow rates, where flow rates similar to a firehose or lower (≤ 2548 L/min; detection probability of 0.23, 95% CI 0.17–0.30) exhibited nearly double the detection probability of a 30‐in. culvert (11,893–110,436 L/min; detection probability of 0.14, 95% CI 0.09–0.22), and eDNA of CDLs was virtually nondetectable in flow rates greater than that of a 30‐in. culvert (> 110,436 L/min; detection probability of 0.03, 95% CI 0.00–0.19; Wald test, *p* < 0.05).

Based on these results, we determined that optimal eDNA sampling conditions for CDLs occurred at night, within 50 m of the origin point, and at lower flow rates. Under optimal sampling conditions (at night and within 50 m of the origin point, Table 4), we estimated that to achieve a cumulative detection probability of > 0.75 (0.79, 0.66–0.89) five 5‐L water samples are required. In comparison, at higher flow rates (11,893–110,436 L/min) seven 5‐L water samples are required to achieve a similar probability of detection (Table 6).

**TABLE 6:** Cumulative detection probabilities for detecting Coeur d'Alene salamanders at occupied sites in Montana, USA, using eDNA under optimal sampling conditions but varying flow rates (see Table 4, line 7 for model).

Sampling conditions	Number of 5 L water samples	Cumulative detection probability
Distance 50 m or less Night samples Flow rate: ≤ 850 L/min	1	0.27 (0.19–0.35)
	4	0.72 (0.57–0.83)
	5	0.79 (0.66–0.89)
	6	0.85 (0.75–0.93)
	7	0.89 (0.78–0.95)
	8	0.92 (0.82–0.97)
	9	0.94 (0.85–0.98)
	10	0.96 (0.88–0.99)
Distance 50 m or less Night samples Flow rate: 2549–110,435 L/min	1	0.20 (0.09–0.31)
	5	0.68 (0.41–0.84)
	6	0.74 (0.46–0.89)
	7	0.79 (0.52–0.92)
	8	0.83 (0.56–0.95)
	9	0.87 (0.61–0.96)
	10	0.90 (0.65–0.97)

## 4 Discussion

We successfully developed an eDNA assay for an elusive salamander species that can be applied in a field setting to determine occupancy. Unlike traditional visual searches, using eDNA allows biologists to systematically evaluate site occupancy with a measured detection probability for both novel and historic sites. We provide a recommended protocol that can be applied to improve our understanding of CDL distribution and thus potential threats to their conservation.

North America supports many plethodontid species, some of which could potentially be amplified by the CDL assay due to genetic similarity at the assay locus; however, none occur within the range of CDLs in Montana. Consequently, our design effort focused on creating an assay specific to CDLs in the ecological context of the species, rather than an assay that would also be completely specific against any other plethodontid. Analysis with eDNAssay suggests that there is one nontarget plethodontid that has a low, but non‐negligible probability of amplifying with the CDL assay, and no plethodontids that will probably be amplified. Further testing in vitro would resolve questions about whether these species' DNA could be amplified or not. None of the species occur in proximity to CDLs, and therefore we assessed the risk of these species causing false positive eDNA amplifications to be extremely low. The results suggest the assay has good generality across the different CDL haplotypes, and good specificity against other western North American amphibian species.

While assay assessment revealed eDNA analysis to be a viable method for detecting CDLs, we found that optimal conditions exist under which to apply eDNA sampling to increase the probability of detecting CDLs. As hypothesized, we found a significant (nearly two‐fold) increase in the probability of detection at night compared to daylight hours. We suspect that this is related to CDL surface activity being higher at night (Nussbaum et al. 1983); CDLs emerge from subterranean spaces at night, potentially making eDNA more available in the surface water for collection than during daylight hours when CDLs are relatively inactive above ground and not in direct contact with surface water. In comparison, Pilliod et al. (2013) found no difference in eDNA concentrations of tailed frogs and Idaho giant salamanders between day and night sampling periods; however, these species were present in surface stream waters throughout the diurnal cycle. Therefore, the increase in detection probability at night of CDLs may be more related to contact with surface water flow than actual activity levels that may impact shedding of genetic material. Pilliod et al. (2014) found that detection of Idaho giant salamanders occurred 2 h after they were introduced to the stream, and eDNA concentration plateaued at 4–5 h. We found that during field surveys CDLs typically emerged after dark (approximately 30 min past sunset) and therefore became “available” to start shedding DNA into surface waters at that time. Like Pilliod et al. (2014), detection probability was low in the first hour after sunset but increased thereafter until about 5 h post‐sunset, beyond which we didn't collect data. Therefore, it is possible that delaying sampling to at least 60 min after sunset could increase detection rate

Determining how far from a suspected site to sample is key when developing a monitoring protocol. Instream sampling with a known population of Idaho giant salamanders and tailed frogs resulted in no decrease in eDNA concentration at 50 m from the animals (Pilliod et al. 2013), suggesting that samples could be taken anywhere within 50 m and yield similar eDNA results. We found that samples taken at various distances within 50 m of the designated origin point yielded similar detection rates; however, we cannot be certain that CDLs were not distributed throughout that 50‐m corridor. For instance, occasionally we found CDLs downstream of the designated origin point, suggesting the designation of origin points, and subsequent assumption that there were few to no CDLs downstream of those origin points, were inaccurate. Therefore, we recommend sampling downstream of, but as close as possible to, the portion of the potential site with the best habitat conditions (fractured rock, moss, persistent moist conditions) to provide the greatest chance of getting a positive detection if CDLs are present.

Flow rate can impact eDNA availability for sampling, as well as the concentration of DNA in the water (Curtis et al. 2021). As suspected, we found that flow rate was negatively correlated with detection probability of CDLs. When flow rate was greater than that of a 30‐in. culvert (> 110,436 L/min), CDLs were virtually undetectable regardless of distance from the origin point. Indeed, CDL DNA was not detected in any of the samples from 2 large stream sites with very high flow rates regardless of season. Conversely, optimal sampling conditions existed when flow rate was less than that of a firehose (> 850). However, 2 seep sites had such low flows, essentially water dripping off a rock face, that collecting sufficient water was challenging and neither site yielded water samples positive for CDL DNA. As expected, flow rate varied among sites and among seasons. Seeps typically exhibited the lowest flow rates and were often only running on the surface during the spring. In comparison, the larger instream waterfall sites had high flow rates in the spring, making eDNA sampling ineffective, and lower flows in the fall, during which eDNA sampling could potentially be effective. Therefore, optimal sampling time is highly dependent on individual site characteristics. In general, we recommend sampling seeps in the spring if surface water may disappear later in the year and sampling sites with high spring flows in the fall when flow rate is lower.

In addition to instream conditions, seasonal CDL surface activity may impact the optimal time of year to sample; however, we did not have enough data to evaluate this potential effect. Previous publications have asserted that CDL surface activities are limited to early summer and fall when conditions are cool and wet (Wilson Jr. and Larsen Jr. 1988; Werner and Reichel 1994); however, surface activities have been reported from as early as March to as late as November, and CDLs may be found on the surface even when their habitat is largely covered in ice (Wilson Jr. and Larsen Jr. 1988). These previous studies also suggest that surface activity is highest within approximately 24–48 h of rain (Wilson Jr. and Larsen Jr. 1988, Werner and Reichel 1994); however, we found no effect of time since last rain on the probability of detecting salamanders, and visual surveys produced many CDL detections regardless of recent weather conditions.

At sites in the Kootenai National Forest, nighttime surface activity of CDLs appeared greatest in early October at seeps that did not experience seasonal loss of surface water flow. For example, during a two‐night period at one site, we observed 56 salamanders and, on several occasions, CDLs were observed walking down wet trails more than 50 m from any known sites. Therefore, it would seem logical that, if more salamanders were active and interacting with the surface water in the fall, then sampling should be conducted in the fall when possible. Regardless, more examination is warranted to determine whether site‐specific patterns of detection probability are driven by seasonal variation in salamander surface activity, stream flow, or other conditions.

Even under optimal sampling conditions, the probability of detecting CDLs with a single 5‐L water sample remains low, such that it is necessary to collect additional water samples to achieve greater confidence in whether CDLs occur at a site. When samples are collected at night and close to a suspected origin point, we found that to achieve a high degree of certainty (> 75%) that CDLs were detected when present, it is necessary to filter 5, 5‐L water samples when the flow rate was a firehose or less, and 7 samples when the flow rate is between a firehose and a 30‐in. culvert. As some sites can be rather large and CDLs may be present throughout the site, we recommend taking these samples at multiple locations throughout the site in relation to available habitat. Collecting spatially duplicated samples in this manner may improve chances of obtaining a sample with eDNA given unknown locations of CDL

In summary, we recommend the following survey protocol: To maximize the probability of detecting CDLs using eDNA analysis at sites where their occupancy is unknown, we recommend sampling at least 60 min after sunset, as close as possible to the downstream extent of the portion of the site with the best habitat conditions, during periods of low water flow. The amount of sampling required at each site will depend on flow rate, with a minimum of 5, 5‐L samples taken at sites with flow rates of ≤ 2548 L/min, and a minimum of 7, 5‐L samples taken at sites with flow rates > 2548 L/min. If a site is relatively large, consider taking the required number of samples at multiple locations throughout the site in relation to potential areas of higher habitat suitability. For streams and seeps that flow in spring and fall, we recommend fall sampling, as there is anecdotal information that suggests CDL surface activity may be higher at this time of year, reserving spring sampling for sites where surface water is absent in the fall.

## 5 Management Implications

The results of this study indicate that eDNA analysis is a viable method to estimate CDL occupancy at both novel and historic sites across their range, and future studies can adopt the assay described and validated here. Previously, biologists were relegated to visually searching potential sites at night under wet and often treacherous conditions, and while salamanders were often observed during night searches, sometimes they were not. In the case of no visual detections, biologists were left with the uncertainty of whether a site was occupied. This new method provides wildlife managers a standardized way to ascertain with a calculated degree of certainty if a site is occupied when visual searches are not possible or fruitful. However, analyzing 5 eDNA samples from each site can be costly, and therefore we recommend conducting visual searches when possible. If visual searches are conducted in conjunction with eDNA sampling, we recommend first collecting water samples for eDNA analysis to avoid site contamination. Then, if a salamander is detected during the visual search, analyzing the water sample is not necessary. Combining the use of visual searches with eDNA sampling greatly improves our ability to determine the distribution of CDLs in Montana and throughout their range, as well as individual site occupancy over time.

## Author Contributions

**Jessica A. Coltrane:** conceptualization (equal), data curation (equal), formal analysis (supporting), funding acquisition (equal), investigation (equal), methodology (equal), project administration (lead), writing – original draft (lead), writing – review and editing (lead). **Torrey Ritter:** conceptualization (equal), data curation (equal), formal analysis (supporting), funding acquisition (equal), investigation (equal), methodology (equal), project administration (equal), writing – original draft (supporting), writing – review and editing (supporting). **Hannah Specht:** conceptualization (equal), data curation (supporting), formal analysis (lead), funding acquisition (supporting), investigation (supporting), methodology (equal), writing – original draft (equal), writing – review and editing (supporting). **Daniel H. Mason:** formal analysis (lead), investigation (equal), methodology (equal), project administration (equal), writing – original draft (equal), writing – review and editing (supporting). **Thomas W. Franklin:** project administration (equal), supervision (equal), writing – review and editing (supporting). **Alissa Anderson:** project administration (supporting), writing – review and editing (supporting).

## Funding

This work was supported by the Montana Fish, Wildlife and Parks.

## Ethics Statement

Our data collection was primarily noninvasive. CDL genomic DNA, as well as DNA later used for in vitro testing against nontarget species, was extracted from tissue samples in accordance with MFWP regulations and in a manner consistent with ethical and permit guidelines. We did not negatively impact individual animals or populations, and we followed the American Society of Mammalogist's guidelines for conducting wildlife research (Sikes et al. 2016, since the Animal care and use committee includes multiple people).

## Conflicts of Interest

The authors declare no conflicts of interest.

## Data Availability

The authors confirm that the data supporting the findings of this study are available within the article (and/or) its Supporting Information.

## Appendix A

Accessions and taxon information from cytb sequences used during initial design of the Coeur d'Alene salamander assay.

**Table:**

Accession	Genus	Species	Name
AY572046.1	*Plethodon*	*idahoensis*	Coeur d'Alene salamander
AY572047.1	*Plethodon*	*idahoensis*	Coeur d'Alene salamander
AY572048.1	*Plethodon*	*idahoensis*	Coeur d'Alene salamander
AY572049.1	*Plethodon*	*idahoensis*	Coeur d'Alene salamander
AY572050.1	*Plethodon*	*idahoensis*	Coeur d'Alene salamander
AY572051.1	*Plethodon*	*idahoensis*	Coeur d'Alene salamander
AY572052.1	*Plethodon*	*idahoensis*	Coeur d'Alene salamander
AY572053.1	*Plethodon*	*idahoensis*	Coeur d'Alene salamander
AY572054.1	*Plethodon*	*idahoensis*	Coeur d'Alene salamander
AY572055.1	*Plethodon*	*idahoensis*	Coeur d'Alene salamander
AY572056.1	*Plethodon*	*idahoensis*	Coeur d'Alene salamander
AY572057.1	*Plethodon*	*idahoensis*	Coeur d'Alene salamander
AY572058.1	*Plethodon*	*idahoensis*	Coeur d'Alene salamander
AY572059.1	*Plethodon*	*idahoensis*	Coeur d'Alene salamander
AY572060.1	*Plethodon*	*idahoensis*	Coeur d'Alene salamander
AY572061.1	*Plethodon*	*idahoensis*	Coeur d'Alene salamander
AY572062.1	*Plethodon*	*idahoensis*	Coeur d'Alene salamander
AY572063.1	*Plethodon*	*idahoensis*	Coeur d'Alene salamander
AY572064.1	*Plethodon*	*idahoensis*	Coeur d'Alene salamander
AY572066.1	*Plethodon*	*idahoensis*	Coeur d'Alene salamander
AY572067.1	*Plethodon*	*idahoensis*	Coeur d'Alene salamander
AY572070.1	*Plethodon*	*idahoensis*	Coeur d'Alene salamander
AY572071.1	*Plethodon*	*idahoensis*	Coeur d'Alene salamander
AY572072.1	*Plethodon*	*idahoensis*	Coeur d'Alene salamander
AY572073.1	*Plethodon*	*idahoensis*	Coeur d'Alene salamander
AY572074.1	*Plethodon*	*idahoensis*	Coeur d'Alene salamander
AY572075.1	*Plethodon*	*idahoensis*	Coeur d'Alene salamander
AY572076.1	*Plethodon*	*idahoensis*	Coeur d'Alene salamander
AY572077.1	*Plethodon*	*idahoensis*	Coeur d'Alene salamander
AY572078.1	*Plethodon*	*idahoensis*	Coeur d'Alene salamander
AY572079.1	*Plethodon*	*idahoensis*	Coeur d'Alene salamander
AY572080.1	*Plethodon*	*idahoensis*	Coeur d'Alene salamander
AY572081.1	*Plethodon*	*idahoensis*	Coeur d'Alene salamander
AY572082.1	*Plethodon*	*idahoensis*	Coeur d'Alene salamander
AY572084.1	*Plethodon*	*idahoensis*	Coeur d'Alene salamander
AY572085.1	*Plethodon*	*idahoensis*	Coeur d'Alene salamander
AY572086.1	*Plethodon*	*idahoensis*	Coeur d'Alene salamander
AY572087.1	*Plethodon*	*idahoensis*	Coeur d'Alene salamander
AY572088.1	*Plethodon*	*idahoensis*	Coeur d'Alene salamander
AY572089.1	*Plethodon*	*idahoensis*	Coeur d'Alene salamander
AY572090.1	*Plethodon*	*idahoensis*	Coeur d'Alene salamander
AY572091.1	*Plethodon*	*idahoensis*	Coeur d'Alene salamander
AY572092.1	*Plethodon*	*idahoensis*	Coeur d'Alene salamander
AY572093.1	*Plethodon*	*idahoensis*	Coeur d'Alene salamander
AY572094.1	*Plethodon*	*idahoensis*	Coeur d'Alene salamander
AY572095.1	*Plethodon*	*idahoensis*	Coeur d'Alene salamander
AY572097.1	*Plethodon*	*idahoensis*	Coeur d'Alene salamander
AY572098.1	*Plethodon*	*idahoensis*	Coeur d'Alene salamander
AY572099.1	*Plethodon*	*idahoensis*	Coeur d'Alene salamander
AY572100.1	*Plethodon*	*idahoensis*	Coeur d'Alene salamander
AY572101.1	*Plethodon*	*idahoensis*	Coeur d'Alene salamander
AY572102.1	*Plethodon*	*idahoensis*	Coeur d'Alene salamander
AY572103.1	*Plethodon*	*idahoensis*	Coeur d'Alene salamander
AY572104.1	*Plethodon*	*idahoensis*	Coeur d'Alene salamander
AY572105.1	*Plethodon*	*idahoensis*	Coeur d'Alene salamander
AY572106.1	*Plethodon*	*idahoensis*	Coeur d'Alene salamander
AY572107.1	*Plethodon*	*idahoensis*	Coeur d'Alene salamander
AY572096.1	*Plethodon*	*idahoensis*	Coeur d'Alene salamander
AY691759.1	*Plethodon*	*vandykei*	Van Dyke's salamander
AY572039.1	*Plethodon*	*vandykei*	Van Dyke's salamander
AY572040.1	*Plethodon*	*vandykei*	Van Dyke's salamander
AY572041.1	*Plethodon*	*vandykei*	Van Dyke's salamander
AY572042.1	*Plethodon*	*vandykei*	Van Dyke's salamander
AY572043.1	*Plethodon*	*vandykei*	Van Dyke's salamander
AY572044.1	*Plethodon*	*vandykei*	Van Dyke's salamander
AY572045.1	*Plethodon*	*vandykei*	Van Dyke's salamander
AY691760.1	*Plethodon*	*vehiculum*	Western redback salamander
JF509398.1	*Plethodon*	*vehiculum*	Western redback salamander
JF509399.1	*Plethodon*	*vehiculum*	Western redback salamander
JF509400.1	*Plethodon*	*vehiculum*	Western redback salamander
AY183763.1	*Plethodon*	*dunni*	Dunn's salamander
EF036633.1	*Ambystoma*	*macrodactylum*	Long‐toed salamander
JX650148.1	*Ambystoma*	*macrodactylum*	Long‐toed salamander
AY691729.1	*Ambystoma*	*gracile*	Northwestern salamander
EF036622.1	*Ambystoma*	*gracile*	Northwestern salamander
EF036665.1	*Ambystoma*	*tigrinum*	Tiger salamander
EF036666.1	*Ambystoma*	*tigrinum*	Tiger salamander
AY734596.1	*Dicamptodon*	*tenebrosus*	Coastal giant salamander
AY734581.1	*Dicamptodon*	*tenebrosus*	Coastal giant salamander
AY691727.1	*Rhyacotriton*	*cascadae*	Cascade torrent salamander
AY691728.1	*Rhyacotriton*	*kezeri*	Columbia torrent salamander
EF036689.1	*Rhyacotriton*	*olympicus*	Olympic torrent salamander
AY627912.1	*Taricha*	*granulosa*	Rough‐skinned newt
KJ536215.1	*Pseudacris*	*maculata*	Boreal chorus frog
KJ536196.1	*Pseudacris*	*regilla*	Pacific tree frog
DQ087514.1	*Ascaphus*	*montanus*	Rocky Mountain tailed frog
AF277327.1	*Ascaphus*	*truei*	Tailed frog
EU552219.1	*Rana*	*aurora*	Northern red‐legged frog
EU708878.1	*Rana*	*cascadae*	Cascades frog
EU708872.1	*Rana*	*pretiosa*	Oregon spotted frog
AY083274.1	*Rana*	*clamitans*	Green frog
AY083270.1	*Rana*	*luteiventris*	Columbia spotted frog
HM563929.1	*Bufo*	*boreas*	Western toad
AY083271.1	*Rana*	*sylvatica*	Wood frog
KF049927.1	*Rana*	*catesbeiana*	American bullfrog
